# Carbopol^®^ 940 Hydrogel Functionalised with Plasma-Activated Water: An Advanced Platform for Controlled ROS Delivery and Antimicrobial Applications

**DOI:** 10.3390/gels12050403

**Published:** 2026-05-07

**Authors:** Alma Neli Hernández-Arias, Benjamín Gonzalo Rodríguez-Méndez, Régulo López-Callejas, Diego Medina-Castro, Antonio Mercado-Cabrera, Rosendo Peña-Eguiluz, Bethsabet Jaramillo-Sierra, Raúl Valencia-Alvarado

**Affiliations:** 1 Instituto Nacional de Investigaciones Nucleares, Carretera México Toluca-La Marquesa s/n, Ocoyoacac 52750, Estado de México, Mexico; alma.arias@utvtol.edu.mx (A.N.H.-A.); regulo.lopez@inin.gob.mx (R.L.-C.); diego.medina@inin.gob.mx (D.M.-C.); antonio.mercado@inin.gob.mx (A.M.-C.); rosendo.eguiluz@inin.gob.mx (R.P.-E.); raul.valencia@inin.gob.mx (R.V.-A.); 2 Dirección de Procesos Alimenticios, Unidad Académica de Capulhuac, Universidad Tecnológica del Valle de Toluca, Lomas de San Juan s/n, Capulhuac de Mirafuentes 52700, Estado de México, Mexico; 3 División de Ingeniería Ambiental, Tecnológico de Estudios Superiores de Tianguistenco, Tecnológico Nacional de México, Carretera Tenango-La Marquesa Km 22, Tianguistenco 52650, Estado de México, Mexico; bethsabet.jaramillo@test.edu.mx

**Keywords:** plasma-activated water (PAW), Carbopol^®^ 940, oxidative drug delivery, reactive oxygen species (ROS) stability, wound healing, diabetic foot ulcer (DFU), non-thermal plasma, antimicrobial resistance (AMR), translational medicine

## Abstract

The antimicrobial resistance crisis necessitates innovative systems for delivering oxidising agents. This study reports the development of a Carbopol^®^ 940 hydrogel functionalised with plasma-activated water (PAW) for the stabilisation and controlled release of reactive oxygen species (ROS). PAW was synthesised using a dielectric barrier discharge (DBD) reactor with continuous flow of water. The hydrogel’s architecture was characterised via SEM and FTIR, revealing a self-organised nanoporous structure (~1433 nm) that acts as a chemical reservoir. This architecture resulted in 100% retention of O_3_ and H_2_O_2_ for 90 min, significantly extending the biological activity window compared with liquid PAW, and maintaining therapeutic concentrations (3 ppm of H_2_O_2_) beyond 45 h. In vitro antibacterial potency against *Escherichia coli* was validated, yielding a clear 25 mm inhibition zone. Subsequently, a clinical proof-of-concept was conducted in a patient with a recalcitrant Wagner Grade 2 diabetic foot ulcer (DFU). The hydrogel as monotherapy—without systemic antibiotics—achieved complete infection remission and full wound closure within 60 days. While this *n* = 1 case demonstrates translational feasibility, further validation through an ongoing controlled clinical trial is required. In conclusion, the PAW–Carbopol^®^ 940 hydrogel is a disruptive, low-cost therapeutic platform that effectively eradicates infection and promotes tissue repair through oxidative eustress, positioning it as a sustainable alternative for the advanced management of complex chronic wounds and regenerative medicine.

## 1. Introduction

The potential of non-thermal plasma (NTP) for cell and tissue treatment is driving rapid growth in research and technological development, generating synergies with areas such as biotechnology, microbiology, and medicine [1,2]. This line of research poses significant technological challenges and fundamental questions about the mechanisms of interaction between plasma and living matter. During the last decade, biomedical applications of plasmas have focused primarily on surface sterilisation and the treatment of materials to control their biocompatibility [3]. However, recent research has extended its use to the treatment of living tissues, including wound healing [4], blood coagulation [5], dermatological applications [6], and other organs [7].

The NTP has been extensively explored for bacterial inactivation [8], demonstrating its effectiveness as a highly non-toxic, residue-free treatment [1]. Plasma, considered the fourth state of matter, is generated by ionising a precursor gas (such as argon, helium, nitrogen, oxygen, or mixtures thereof) through an electric field, resulting in a medium composed of ions, electrons, photons, and neutral particles. The efficiency of this process depends intrinsically on the geometry and arrangement of the electrodes in the reactor [9]. The key to its biomedical applications lies in the generation of reactive oxygen species (ROS) and reactive nitrogen species (RNS) at atmospheric pressure and ambient temperature. These species, which include ozone (O_3_), hydrogen peroxide (H_2_O_2_), the hydroxyl radical (^•^OH), and the superoxide anion (O_2_^−^), actively participate in cell physiology and bacterial lysis [8]. In direct applications, the effects of ultraviolet radiation, electric fields, and charged particles also converge.

Although the direct application of plasma—generated by dielectric barrier discharge (DBD) or plasma jets (APPJ)—is effective for sterilisation, indirect application using plasma-activated water (PAW) is emerging as a promising alternative. In this process, the plasma transfers the ROS generated in the gas phase to the liquid phase, modifying the physicochemical properties of the water, which can degrade organic contaminants and eliminate microorganisms [10].

Furthermore, hydrogels are hydrophilic polymer networks with a three-dimensional structure, formed by cross-linked polymer chains. This cross-linking confers insolubility to the structure and allows the absorption of large volumes of fluid (often more than 100 times its dry weight) through ionic interactions and hydrogen bonds [11]. This absorption capacity and the subsequent release of substances trapped in its pores (via diffusion and convection) depend on the network’s properties, such as porosity and diffusion coefficient [12]. Due to their versatility, hydrogels are used across sectors from agriculture to biomedicine, serving as controlled-release systems for therapeutic agents [13]. For their medical and therapeutic use, it is imperative that they meet requirements for biocompatibility, stability against degradation, and appropriate mechanical properties.

Despite the efficacy of plasma, its direct application can be limited, and the use of liquid antimicrobial agents (as PAW) lacks a mechanism for sustained and localised delivery of ROS to the application site [14]. Given the antimicrobial resistance crisis, which demands alternatives to antibiotics, stabilising ROS in a topical matrix represents a promising solution.

This study demonstrates the feasibility of using Carbopol^®^ 940 (polyacrylic acid or carboxyvinyl polymer) from Gardenia Naturals Labs (Guadalajara, Mexico) hydrogel [15] as a polymeric matrix to encapsulate, stabilise, and sustainably release chemical species generated in PAW. A functionalized hydrogel was formulated, and its antimicrobial efficacy was evaluated in vitro against *Escherichia coli* (*E. coli*), a model Gram-negative pathogen. While various studies have explored plasma-activated hydrogels using materials such as hydroxyethyl cellulose, carbomer 940, acryloyldimethylammonium taurate/VP copolymer [16], gelatin [17], or alginate, [18], the present study introduces three distinctive features that have not been previously reported. First, PAW is produced by a DBD reactor in continuous flow, obtaining a large quantity of PAW in just a few minutes of treatment (100 mL/min). Second, in our process, the only working gas is oxygen, avoiding the need for other costly gases such as argon or helium. Third, beyond in vitro testing, our study incorporates a translational clinical observation often absent from current literature: namely, a patient with a recalcitrant Wagner Grade 2 diabetic foot ulcer treated with PAW–Carbopol^®^ 940 hydrogel as monotherapy. This clinical proof-of-concept is intended to fill a critical translational gap that is largely absent in the current literature. Collectively, these features distinguish our work from earlier reports and position the PAW–Carbopol^®^ 940 hydrogel as a promising candidate for controlled ROS delivery in chronic wound management.

## 2. Results and Discussion

### 2.1. Physicochemical and Kinetic Characterisation of ROS in PAW

The chemical reactivity induced by the plasma in the water was evaluated by monitoring pH and quantifying long-lived oxidising species. The precursor distilled water had a pH of 5.4 ± 0.2. After exposure to the DBD reactor, all samples showed systematic acidification, with final pH values from 4.2 to 5.1. The observed reduction in pH (from 5.4 to the 4.2–5.1 range) is primarily attributed to the formation of nitric and nitrous acids (HNO_3_ and HNO_2_) through nitrogen fixation in the air–plasma interface [19,20]. While this study focuses on the quantification of O_3_ and H_2_O_2_ as the dominant oxidising agents, the presence of these nitrogen-derived species (nitrates and nitrites) is a key factor in establishing the acidic environment that enhances the stability and antimicrobial synergy of the hydrogel. Although the generation and influence of RNS are acknowledged [21,22], their quantitative evaluation was outside the scope of this study, which represents a current limitation. Future studies will involve the specific quantification of these RNS to further refine the chemical profile of the platform and to elucidate their precise role in the observed therapeutic effects.

**Figure 1:**
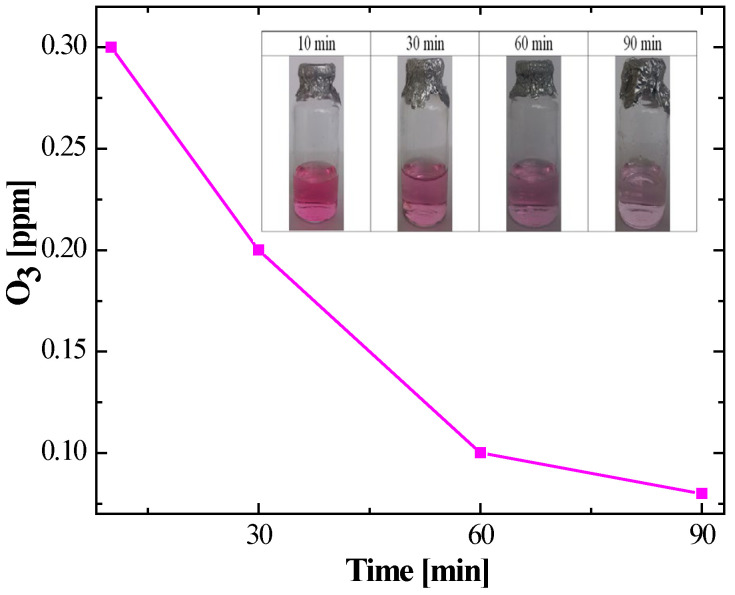
Concentration of dissolved ozone in PAW was measured for 90 min after treatment, showing a progressive decrease from 0.3 ppm to lower levels.

**Figure 2:**
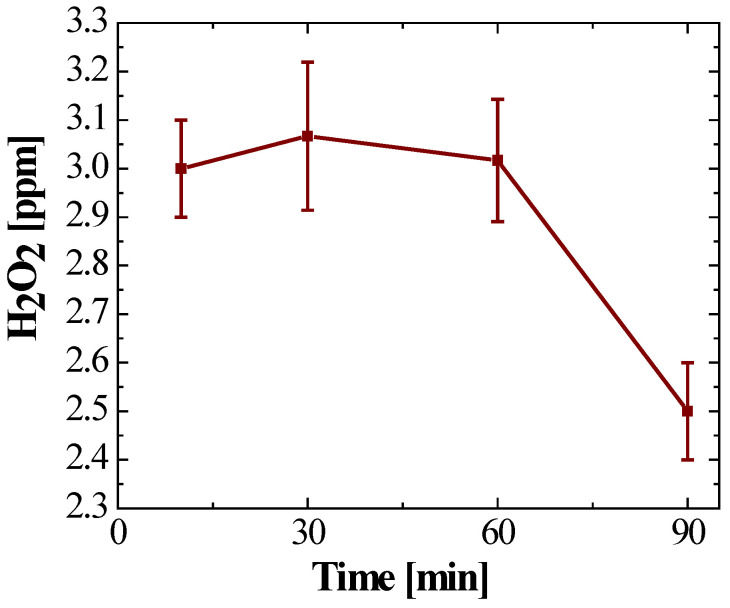
Time profile of hydrogen peroxide dissolved in PAW, with a stable concentration close to 3 ppm for 60 min, followed by a slight decrease to approximately 2.5 ppm at 90 min.

The stability of ROS in the PAW is presented next. The kinetic profiles revealed differentiated behaviours:

- Specifically, ozone (O_3_) exhibited an initial maximum concentration of 0.3 ppm. However, temporal monitoring showed accelerated degradation kinetics. As illustrated in Figure 1, ozone levels declined steadily, approaching the method’s detection limit 90 min post-activation. This behaviour is attributed to the gas’s low solubility and its rapid decomposition in aqueous media.
- In contrast, hydrogen peroxide (H_2_O_2_) exhibited significantly greater stability. The initial concentration was 3.0 ppm, remaining constant for the first 60 min. Subsequently, a moderate decay phase was observed, registering a residual concentration of 2.5 ppm at 90 min (Figure 2). This represents a retention of 83.3% of the initial load, confirming H_2_O_2_ as the most persistent oxidising species in the liquid system.

### 2.2. Structural, Morphological, and Rheologica Characterisation of the Hydrogel

**Figure 3:**
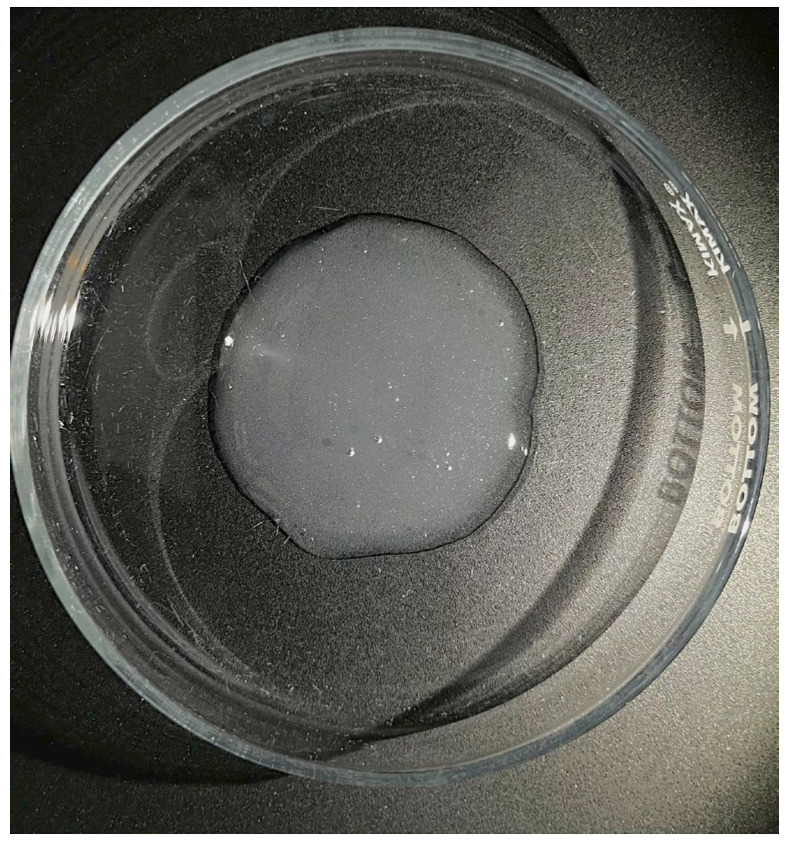
Image of the hydrogel formulated with Carbopol^®^ 940 and PAW, showing a crystalline white colour and homogeneity of the gel mass, with the presence of dispersed microbubbles.

The sol–gel phase transition confirmed the formation of a stable, functional polymer matrix. The final hydrogel exhibited a dynamic viscosity of 1.38 Pa·s and a pH of 5.5 ± 0.2. These parameters confer ideal rheological behaviour for topical adhesion and retention in the wound bed. They also ensure biocompatibility aligned with pharmacopoeial standards for treating chronic dermal lesions. Organoleptically, the system presented a white, crystalline appearance and a homogeneous texture (Figure 3). Uniformly dispersed microbubbles characterise its texture. This macroscopic morphology resulted from the agitation and homogenization of the PAW within the polymer network.

**Figure 4:**
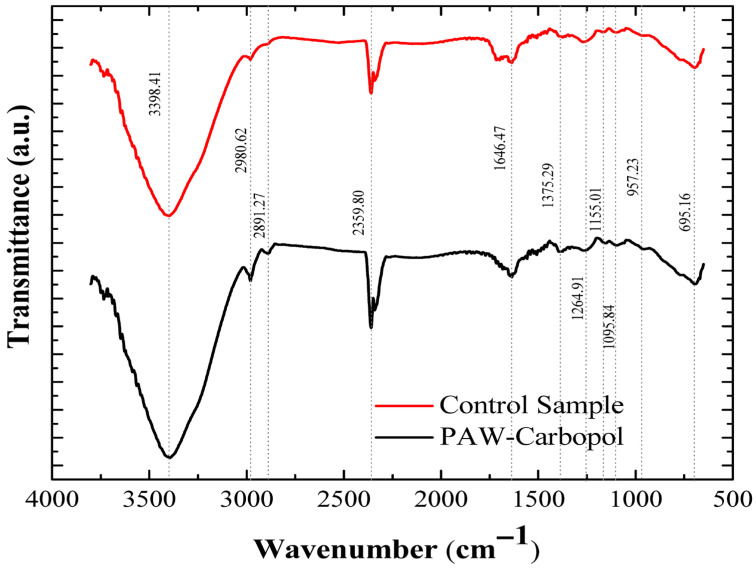
FTIR spectra of the Carbopol^®^ 940 hydrogel. Spectral comparison between the control (red) and the hydrogel functionalized with PAW (black).

To validate the structural integrity of the polymer after interaction with the oxidising species of PAW, an analysis was performed using Fourier Transform Infrared Spectroscopy (FTIR). The spectra of the control sample and the functionalized hydrogel, in the 4000–500 cm^−1^ region, are shown in Figure 4. In the Carbopol^®^ 940 spectrum, a broad band is observed between 3100 and 3550 cm^−1^, centred at approximately 3400 cm^−1^, associated with the stretching vibrations of the hydroxyl (-OH) groups of the carboxylic acids. The intense band at 1646.47 cm^−1^ corresponds to the stretching of the carbonyl (C=O) group. Notably, in the functionalized hydrogel, intense signals were observed at ~1560 cm^−1^ and ~1400 cm^−1^, attributed to the asymmetric and symmetric stretching vibrations of the carboxylate group (-COO^−^), respectively. This spectral change confirms the polymer’s ionisation and the formation of a three-dimensional network, without evidence of main-chain degradation. Likewise, the signal at 2359.80 cm^−1^, associated with atmospheric or trapped CO_2_, and the C-H vibrations near 2900 cm^−1^ maintain consistency, confirming the chemical stability of the matrix in the plasma’s oxidising environment.

This spectral change provides direct evidence of the effective ionisation of the carboxyl groups in Carbopol^®^ 940, leading to the formation of a polyacrylate network. The preservation of absorption signals in the “fingerprint” region (1000–1500 cm^−1^), and the absence of additional bands or significant shifts from chain cleavage, confirm that ROS in PAW do not compromise the polymer’s fundamental chemical integrity. These findings reaffirm the matrix’s robustness as a delivery vehicle, maintaining structural functionality even in a highly oxidising environment.

**Figure 5:**
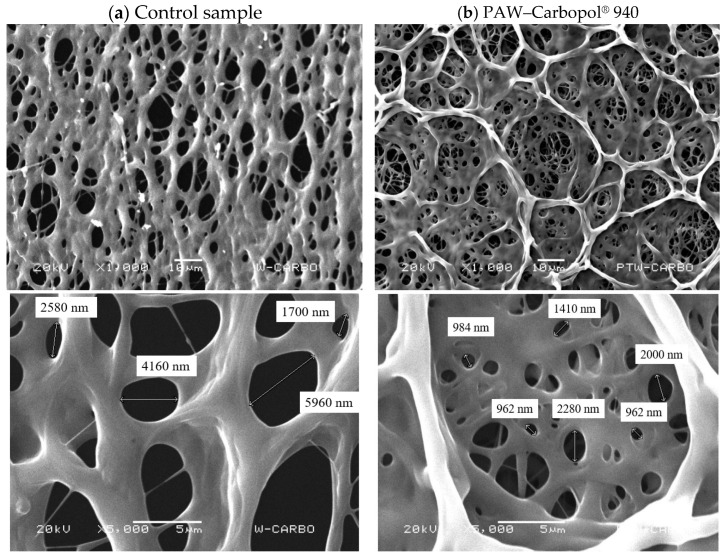
Morphological characterisation of the nanoporous architecture via SEM. Micrographs of the lyophilised matrices illustrate the structural transition induced by PAW. (**a**) Control sample: Prepared with distilled water, exhibiting a heterogeneous, less-defined porosity with irregular polymer wall distribution. (**b**) PAW–Carbopol^®^ 940: Functionalised hydrogel displaying a highly organised, three-dimensional network of interconnected pores with an average diameter of 1433 nm. The superior structural ordering and geometric homogeneity in (**b**) suggest that the reactive species and increased ionic strength of PAW act as kinetic templates, modulating the electrostatic repulsion and crosslinking density of the polyacrylate chains during the sol–gel transition. The labels X1,000 and X5,000 should be read as X1000 and X5000, respectively.

The structure was characterised by Scanning Electron Microscopy (SEM) (Figure 5). Micrographs revealed a highly organised three-dimensional morphology in the functionalised samples. Specifically, the PAW–Carbopol^®^ 940 hydrogel (Figure 5b) exhibited a network of interconnected pores with an average diameter of 1433 nm. This structural refinement suggests a significant rearrangement of the polymer chains, driven by the increased ionic strength of the plasma-activated medium. In contrast, the control samples prepared with distilled water (Figure 5a) displayed a heterogeneous porosity and a less-defined, amorphous architecture.

Detailed analysis of Figure 5b indicates that plasma-derived species induce a kinetically controlled self-assembly during the sol–gel transition. The reactive species and ionic by products of PAW modulate the medium’s ionic strength, promoting a more uniform electrostatic repulsion between the Carbopol^®^ 940 chains during neutralisation with triethanolamine (TEA). This regular architecture is a key functional determinant, as it creates physical confinement that limits the diffusion of ROS. Consequently, ROS exhibits increased residence time and enhanced chemical stability within the matrix. Furthermore, this interconnected porosity is clinically crucial; it facilitates efficient exudate management in diabetic foot ulcers while enabling the controlled, sustained release of reactive species into the wound bed, maintaining a therapeutic oxidative environment.

### 2.3. Stability of Reactive Species in the Hydrogel

The temporal stability of ROS in the polymer matrix showed a superior protective effect compared to the conventional liquid phase. Ozone (O_3_) maintained a steady concentration of 0.3 ppm over 90 min, indicating 100% retention. Hydrogen peroxide (H_2_O_2_) likewise remained at 3.0 ppm throughout the study. The hydrogel exhibited negligible standard deviation (SD), confirming high reproducibility of the encapsulation method and validating the Carbopol^®^ 940 matrix in mitigating ROS autocatalytic degradation.

Follow-up measurements at 21 and 45 h post-formulation revealed that while O_3_ concentrations decreased to 0.2 ppm, H_2_O_2_ levels remained remarkably stable at 3 ppm. This indicates that H_2_O_2_ acts as the primary long-term oxidant, ensuring a sustained therapeutic effect beyond the initial 90 min window.

### 2.4. Antimicrobial Efficacy and Bacterial Inactivation Kinetics

**Figure 6:**
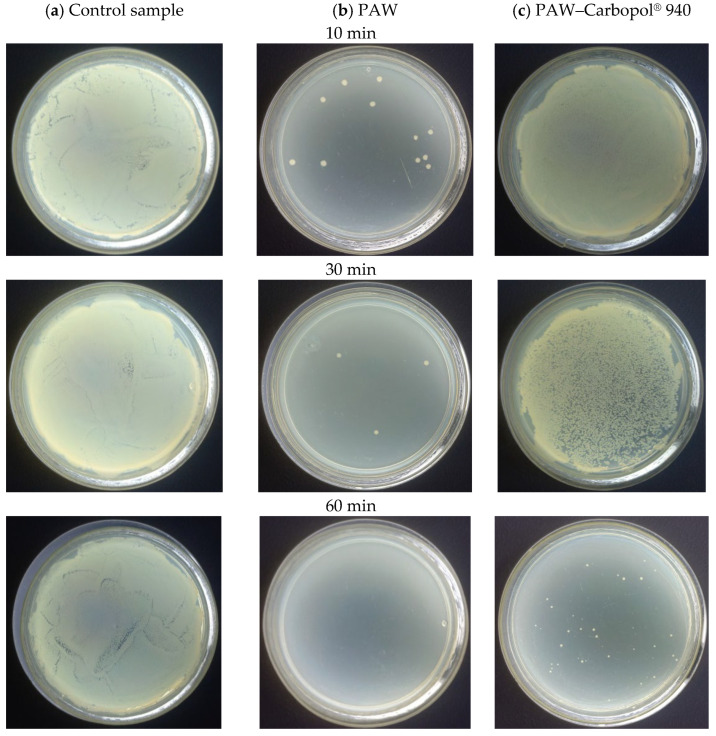

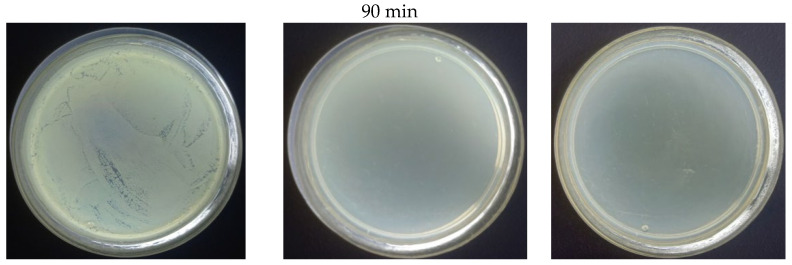
Culture images on LB agar for *E. coli* (10^6^ CFU/mL): (**a**) untreated control; (**b**) after exposure to PAW; (**c**) after exposure to the PAW–Carbopol^®^ 940 hydrogel, for contact times of 10 to 90 min.

Microbiological assays confirmed that Carbopol^®^ 940 hydrogel with PAW is potently bactericidal against *Escherichia coli*. Control groups with base matrix and distilled water showed constant bacterial viability at 10^6^ CFU/mL over 90 min (Figure 6). This stable bacterial population confirms that there is no intrinsic toxicity from the polymer or TEA. The antimicrobial efficacy comes only from reactive species stabilised within the nanoporous structure.

**Figure 7:**
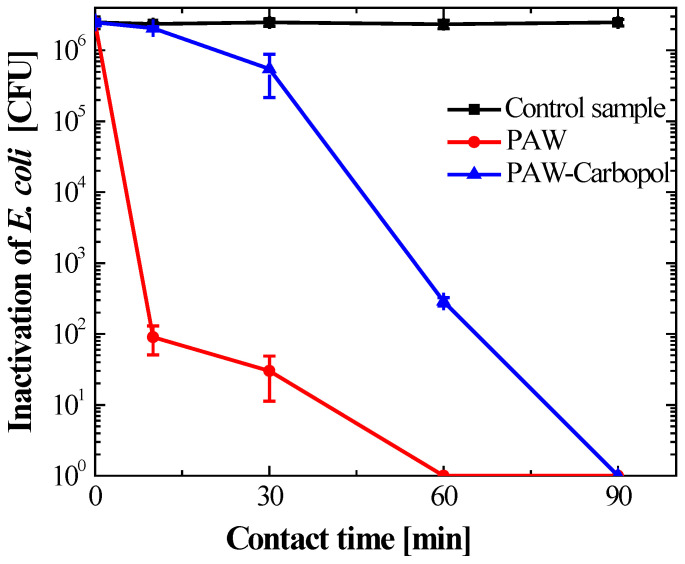
Bacterial inactivation kinetics. The dots represent mean colony-forming unit (CFU/mL) counts of *Escherichia coli* after exposure to the agent. For PAW, a burst effect reduced viability to 0 at 60 min. In contrast, PAW–Carbopol^®^ 940 inactivation was gradual but sustained. Data are shown as mean ± SD from three independent biological replicates (*n* = 3).

**Figure 8:**
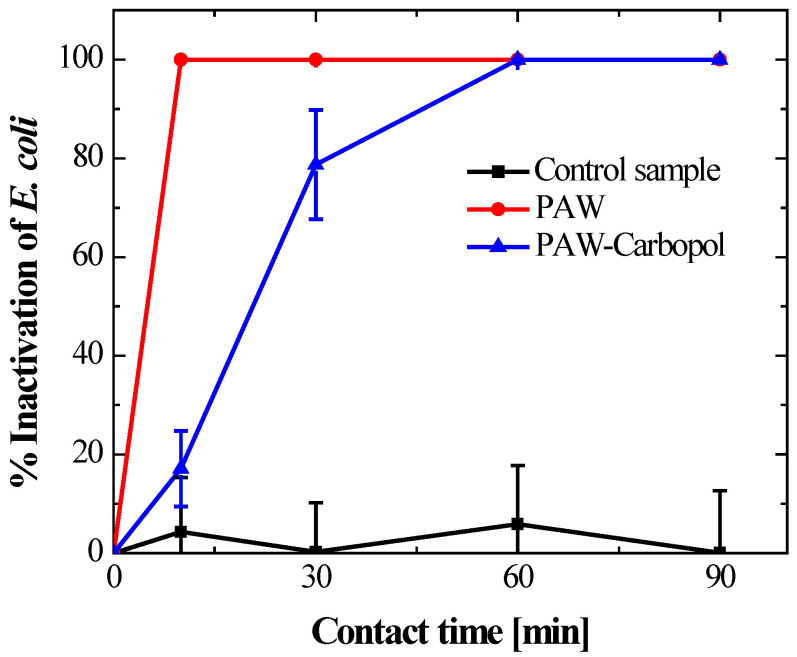
Bacterial inactivation profile and sustained release kinetics of the PAW–Carbopol^®^ 940 hydrogel. Temporal evolution of the microbial load of *E. coli* in contact with the functionalized matrix. The curve illustrates the complete elimination of viability at 90 min. These kinetics, compared with the liquid phase, validate the polymer matrix’s stabilisation and controlled delivery capacity. Dots represent the mean ± SD (*n* = 3).

**Figure 9:**
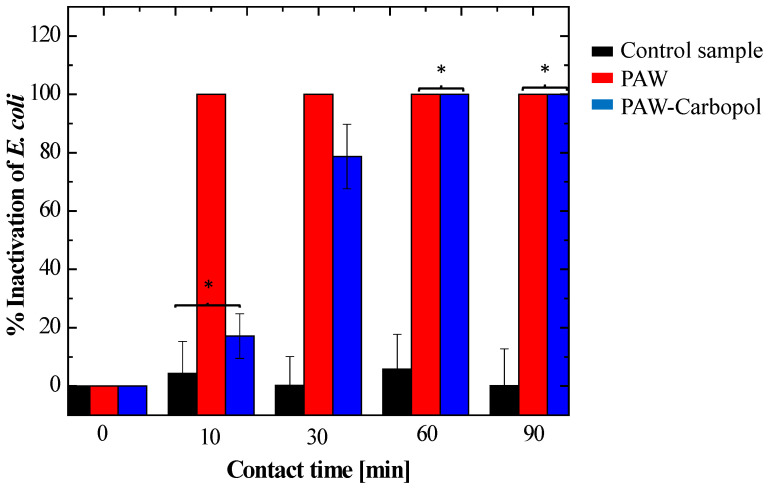
Comparison of *E. coli* inactivation efficiency between the control, PAW, and PAW–Carbopol^®^ 940 hydrogel. Convergence of hydrogel efficacy with the liquid treatment is observed toward the end of the trial. Data represent the mean ± standard deviation (*n* = 3). * indicates that there are not significant differences (*p* > 0.05).

Building on previous culture observations, the activated systems showed distinct inactivation profiles. Bacterial viability data (Figure 7 and Figure 8) showed a statistically significant difference (*p* < 0.05) in PAW versus hydrogel kinetics. PAW produced faster bactericidal effects, reaching total CFU reduction at 60 min (Figure 9), while the hydrogel inactivation was more gradual.

The PAW–Carbopol^®^ 940 hydrogel showed gradual, sustained inactivation. Bacterial levels declined progressively from 10 min, reaching full inactivation at 90 min (Figure 8). This timing difference relative to the liquid phase supports the controlled-release mechanism and the stability of ROS within the nanoporous structure. ANOVA confirmed the Carbopol^®^ 940 matrix lacks intrinsic bactericidal activity; no significant (*p* > 0.05) differences in bacterial viability were seen between the initial inoculum and the control group during the study. Thus, the antimicrobial effect is solely due to modulated ROS release.

As evidenced in the multivariate comparison (Figure 9), although no significant differences were observed between the control and the hydrogel at 10 min, the hydrogel’s efficacy converged with that of the liquid PAW at 60 and 90 min. This successful transition from a burst release to an extended-release profile demonstrates that the polyacrylate matrix acts as a diffusive barrier, modulating the flow of ROS to the bacterial interface. This phenomenon not only prolongs antimicrobial activity but also optimises the bioavailability of the oxidising species, a critical factor for the treatment of chronic wounds where persistent therapeutic action is required. Taken together, these findings position the PAW–Carbopol^®^ 940 hydrogel as an advanced oxidising species delivery system with high efficacy against clinically relevant Gram-negative pathogens.

**Figure 10:**
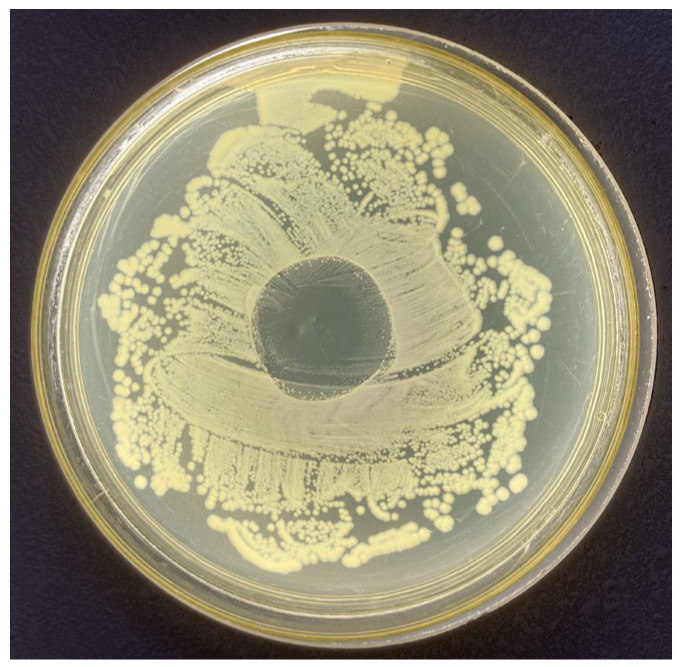
Inhibition zone assay of the PAW–Carbopol^®^ 940 hydrogel against *E. coli*. The clear halo demonstrates the effective diffusion and microbicidal potency of the stabilised ROS.

To further visualise the biological potency and the diffusion capacity of the reactive species from the polymer matrix, an inhibition zone assay was performed (Figure 10). The hydrogel produced a clear and well-defined inhibition halo against *E. coli*, with an average diameter of 25 mm. This macroscopic evidence confirms that the ROS stabilised within the nanoporous structure effectively migrate into the surrounding medium, creating a sterile area where bacterial growth is completely inhibited. This observation provides a direct mechanistic link between the controlled-release kinetics observed in the liquid assays and the platforms potential for decontaminating the wound bed in a clinical setting.

The structural findings correlate with the exceptional chemical stability of the platform. Specifically, our results regarding the 90 min retention of O_3_ and H_2_O_2_ at 100% concentration levels represent a significant improvement over liquid PAW, which typically shows rapid degradation of short-lived species. Similar to Chen et al. [16], in which a plasma-activated hydrogel was developed for the storage and controlled slow-release of RONS, our PAW–Carbopol^®^ 940 system acts as a sustained-release reservoir. The SEM analysis confirms that the interaction between plasma-generated species and the polymer induces a specific nanoporous morphology (~1433 nm) that optimises gas–liquid-solid diffusion, providing a superior therapeutic window for chronic wound treatment.

Furthermore, the therapeutic superiority of the PAW-functionalized hydrogel compared to conventional chemical solutions (such as standalone H_2_O_2_ or O_3_ mixtures) is rooted in the synergistic action of the generated RONS [23]. While H_2_O_2_ provides a sustained oxidative stress, the presence of trace nitrogen species and transient species generated during plasma activation enhance the permeability of bacterial membranes, leading to accelerated inactivation kinetics. Specifically, the synergistic effect within PAW is not a mere summation of individual components, but rather a dynamic system where short-lived reactive species (such as the hydroxyl radical ^•^OH) act as primary stressors to disrupt and sensitise the bacterial membrane, allowing long-lived species (H_2_O_2_ and ozone-derived species) to penetrate and exert bactericidal action more efficiently [24]. This multi-species approach not only increases antimicrobial efficacy but also prevents the development of bacterial resistance. Additionally, the use of plasma technology offers a sustainable, point-of-care production method that eliminates the need for stabilised chemical precursors, positioning this platform as a viable alternative for the management of chronic wounds.

While the 100% retention of short-lived species such as O_3_ was monitored over a 90 min window, the long-term therapeutic efficacy of the dressing (24–72 h) is likely sustained by the higher stability of H_2_O_2_ and nitrogen-derived RNS within the polymer network. This initial concentration of ROS is critical for the destabilisation of bacterial biofilms, while the subsequent sustained release of long-lived species maintains a bacteriostatic environment during clinically relevant application timeframes [25]. Given that significant concentrations are still present at 90 min (H_2_O_2_ at 2.5 ppm, O_3_ at 0.3 ppm), it is plausible that the release will continue for additional hours, although this will need to be evaluated.

### 2.5. Preliminary Clinical Proof-of-Concept: A Single-Case Study

To validate the biological efficacy of the functionalized hydrogel in a complex clinical setting, the evolution of a recalcitrant diabetic foot ulcer (DFU) was evaluated in a 64-year-old male patient. The clinical study was conducted in accordance with the Declaration of Helsinki and was approved by the Institutional Research Ethics Committee of the ISSEMYM Medical Centre (Protocol Code: UEeIM: 314/26; approved on 9 April 2026). Furthermore, the protocol is registered at ClinicalTrials.gov (Identifier: NCT07541196). Informed consent was obtained from the subject prior to the intervention. The lesion, classified as Grade 2 on the Wagner scale, had an elliptical morphology measuring 3.0 cm × 2.5 cm in the plantar region. Clinically, the ulcer exhibited signs of persistent bacterial biofilm, atonic borders, and a bed with scant granulation tissue. It is imperative to emphasise that the protocol was performed under monotherapy, without systemic or topical antibiotics, to isolate the intrinsic therapeutic effect of the PAW–Carbopol^®^ 940 hydrogel. It is important to note that the clinical observation presented here (*n* = 1) is strictly a preliminary finding and a proof-of-concept for the translational potential of the platform. While it demonstrates potential value for wound care, further controlled clinical trials—currently in the recruitment phase—are required to establish generalised therapeutic efficacy and statistical and clinical significance.

**Figure 11:**
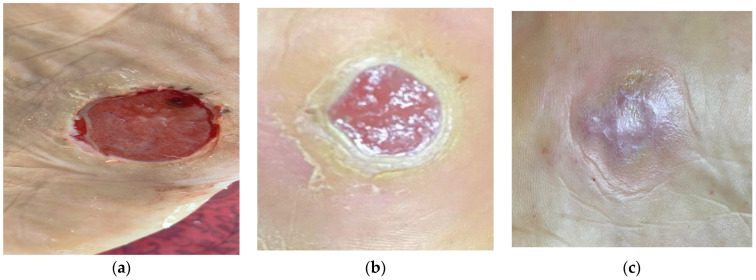
Clinical evolution of a diabetic foot ulcer (DFU) treated with PAW–Carbopol^®^ 940 hydrogel. (**a**) Baseline (Day 0): The lesion presents devitalized tissue, fibrin, and active exudate, indicating chronicity and infection. As treatment progresses, (**b**) Granulation phase (Day 30): the ulcerated area is reduced, and active borders with highly vascularized granulation tissue become visible. Finally, (**c**) Clinical resolution (Day 60): the wound fully closes with total epithelialization, restoring skin barrier integrity.

Tissue repair dynamics were documented in three critical stages (Figure 11):

(i) Baseline (Day 0): The ulcer had a surface area of approximately 7.5 cm^2^, characterised by the presence of slough and seropurulent exudate. These conditions are indicative of a stagnant chronic inflammatory phase and a high microbial load that prevented wound progression.
(ii) Proliferative and granulation phase (Day 30): After four weeks of treatment, an effective transition to the proliferative phase was observed. The lesion showed a significant centripetal reduction in circumference. The replacement of devitalized tissue with highly vascularized granulation tissue was evident, along with complete remission of local infection signs, suggesting that the hydrogel successfully disrupted the biofilm architecture. The successful transition from a stagnant, infected state to a highly vascularized granulation tissue indicates that the PAW-functionalized hydrogel effectively compromised the bacterial biofilm. Despite the inherent resistance of polymicrobial biofilms typical of DFUs, the sustained release of a synergistic ROS cocktail from the nanoporous matrix (~1433 nm) appears to achieve sufficient penetration depth to disrupt the extracellular polymeric substance (EPS) and eliminate protected bacterial colonies. This clinical outcome provides robust evidence of the platform’s anti-biofilm potential, surpassing the limitations of standard planktonic assays.
(iii) Resolution and complete epithelialization (Day 60): At the end of the observation period, the skin barrier was fully restored. Complete wound closure was characterised by concentric epithelialization and organised tissue remodelling, with no evidence of hypertrophic fibrosis or pathological scarring.

This clinical success directly correlates with the controlled-release kinetics. The matrix’s ability to act as a sustained-release ROS delivery system maintained a constant submaximal oxidative pressure in the wound bed. This microenvironment not only eradicated the pathogenic microbiota but also acted as a redox-hormesis stimulus, in which controlled concentrations of reactive species promote cell signalling, fibroblast proliferation, and accelerated angiogenesis.

Regarding biological safety, although specific in vitro cytotoxicity assays were not performed in this initial stage, the absence of adverse local reactions (irritation, erythema, or necrosis) in the healthy perilesional skin during the 60-day treatment provides preliminary evidence of the hydrogel’s biocompatibility. The maintenance of a eudermic pH of 5.5, together with the controlled delivery of ROS concentrations, likely operates within a therapeutic window that avoids acute oxidative damage to healthy eukaryotic cells while effectively eliminating the prokaryotic load. Furthermore, the literature shows that low concentrations (<100 μM) of H_2_O_2_ (equivalent to 3.4 ppm) are not toxic to fibroblasts after short exposures (<24 h) [26], and for epithelial cells this value ranges from 100 to 300 μM (3.4 to 10.2 ppm) [27]. Higher concentrations of H_2_O_2_ may result in cytotoxicity. Nevertheless, cell viability assays using human keratinocytes and fibroblasts are required to fully confirm the biocompatibility of the PAW–Carbopol^®^ 940 hydrogel.

It is important to note that the clinical results presented here correspond to a single-case preliminary study (*n* = 1). While the complete healing of the Wagner Grade 2 ulcer is highly encouraging, these findings are intended solely as a proof-of-concept for the hydrogel’s translational potential. Due to the lack of a control group and the limited sample size, these results are not statistically significant and require further validation through controlled clinical trials with larger cohorts.

### 2.6. Discussion

This research establishes the successful functionalization of a hydrogel with plasma-activated water (PAW), generated using a dielectric barrier discharge (DBD) system, by stabilising it in a Carbopol^®^ 940 polymer matrix. The quantification of ozone (O_3_) and hydrogen peroxide (H_2_O_2_) as the main reactive oxygen species (ROS) in the PAW confirms the efficient formation of oxidising agents, which are essential for antimicrobial activity. The primary objective was to transition the unstable PAW to a controlled-release system, resulting in a functionalized hydrogel [28] that exhibits high and sustained bactericidal activity against *Escherichia coli*.

The difference in inactivation kinetics between liquid PAW and the hydrogel highlights the critical role of polymer support in chemical reactivity. Due to its species’ high ionic mobility, liquid PAW undergoes rapid and extensive oxidation. In contrast, the Carbopol^®^ 940 matrix serves as a kinetic regulator: its nanoporous structure confines ROS, protecting them from premature degradation and slowing their release. This prolonged, controlled ROS contact with pathogens prevents sudden oxidative stress in healthy tissue and maintains constant bactericidal pressure in clinical settings.

The observed acidification (from 5.4 to a range of 4.2 to 5.1) is a direct result of the formation of nitrous and nitric acids [19,20]. This pH drop is explained by the solvation of acidic species at the gas–liquid interface. Mainly, these are nitric acid (HNO_3_) and nitrous acid (HNO_2_), which are derived from atmospheric nitrogen fixation. Hydronium ions (H_3_O^+^) are also generated from water dissociation [29,30]. The stable pH decrease is a key indicator of efficient mass transfer and the build-up of long-lived chemical species. These species (O_3_ and H_2_O_2_) are later confined within the polymer network. This pH reduction is a crucial factor that, in synergy with ROS, enhances the bactericidal activity of the system [31,32]. This phenomenon correlates with the interaction of plasma with residual air in the reactor, where the solvation of nitrogen oxides (NO_x_) generates an acidic environment that compromises the integrity of the bacterial cell wall. From a mechanistic perspective, this acidification facilitates the diffusion of ROS across the plasma membrane, increasing intracellular oxidative stress through lipid peroxidation [33,34].

The quantification of O_3_ (0.3 ppm) and H_2_O_2_ (3.0 ppm) revealed contrasting kinetic profiles. The rapid decomposition of ozone, which fell to undetectable levels within 90 min, is consistent with the literature highlighting its short half-life and high reactivity in aqueous media [35,36]. Conversely, the relative stability of H_2_O_2_ suggests its role as the predominant long-term oxidising agent [37,38]. However, the microbicidal potency of PAW lies in the synergistic action of a pool of short- and long-lived ROS (including O_2_^−^, ^•^OH, ^1^O_2_), which contribute to oxidative damage and are essential for immediate inactivation [39,40]. Importantly, in these systems, bactericidal activity correlates more closely with the concentrations of stored reactive species than with absolute pH values, emphasising the crucial role of ROS in the inactivation mechanism [41].

#### 2.6.1. Stabilisation and Role of the Polymer Matrix

The main critical challenge of PAW technology—its intrinsic instability [42]—was successfully addressed by using the Carbopol^®^ 940 matrix, a synthetic polymer commonly used as a gelling agent. Experimental analysis demonstrated 100% retention of the O_3_ (0.3 ppm) and H_2_O_2_ (3.0 ppm) concentrations over a 90 min period. These findings are fundamental, as Figure 1 and Figure 2 confirm that confinement within the nanoporous structure of Carbopol^®^ 940 mitigates accelerated decay and outgassing kinetics. This mitigation is attributed to a steric hindrance effect and a drastic reduction in the gas diffusion coefficient within the polymer matrix. By increasing dynamic viscosity and consolidating a network of interconnected pores, the matrix acts as a chemical reservoir, protecting oxidising species from recombination and atmospheric interaction, thus ensuring prolonged therapeutic availability.

FTIR results (Figure 4) support this stability by confirming the chemical transition of carboxyl (-COOH) to carboxylate (-COO^−^) groups after TEA (triethanolamine) neutralisation. This structural change not only induces gelation through electrostatic repulsion but also creates an ionic microenvironment that protects ROS from autocatalytic decomposition.

This exceptional stabilisation is attributed to two fundamental mechanisms:

(i) Physical restraint and confinement: The nanoporous microstructure, with an average pore diameter of 1433 nm as determined by SEM (Figure 5), minimises the molecular diffusion rate and prevents O_3_ volatilisation toward the air interface. It is noteworthy that the structural organisation observed in the PAW–Carbopol^®^ 940 hydrogel is superior to that of the control, suggesting that reactive species actively influence the polymer network conformation during crosslinking. These species likely act as ionic tempering agents, organising the polyacrylate chains into a more defined and stable architecture [43].
(ii) Low-reactivity medium and chemical protection: The specific interaction between carboxylate groups and ROS reduces the consumption of the latter through quenching or unwanted deactivation processes [44].
(iii) The final physicochemical characterisation, with a pH (acidity level) of 5.5 and a dynamic viscosity of 1.38 Pa·s, validated the system’s suitability for prolonged topical applications [45]. Furthermore, the indirect activation method employed in this study prevented matrix liquefaction, a common phenomenon in direct plasma treatment caused by the disruption of the polymer’s hydrogen bonds [8]. Overall, Carbopol^®^ 940 provides a safe, stable, and biocompatible (compatible with living tissue) delivery platform optimised for clinical use [46].

#### 2.6.2. Modulation of Antimicrobial Activity and Clinical Evidence

Before clinical evaluation, it was shown that the bactericidal effect arises solely from the ROS confined within the matrix. Control assays showed that the base polymer and the neutralising agents (TEA) do not inactivate *E. coli* (Figure 6). This confirms that the mechanism of action is purely oxidative [47].

The proposed microbicidal mechanism for the PAW–Carbopol^®^ 940 hydrogel is based on sustained oxidative stress. The polyacrylate architecture modulates the gradual diffusion of ROS into the bacterial cell wall, inducing membrane lipid peroxidation and the oxidation of structural proteins. This damage alters cell permeability, facilitating the translocation of oxidising species to the cytosol, where they cause nucleic acid degradation and massive enzyme inactivation. This controlled delivery is crucial, as it ensures the eradication of pathogens without exceeding acute cytotoxicity thresholds in surrounding healthy tissue [48,49].

Kinetic profiles showed that PAW achieves complete bacterial elimination in 60 min, producing a burst effect, while the hydrogel formulation extends this process to 90 min. This confirms the matrix (the hydrogel structure) acts as a sustained-release system [50,51] that allows gradual and thorough disinfection [52].

The translational impact of this sustained-release formulation is demonstrated by the complete healing of a stubborn diabetic foot ulcer within 60 days, achieved with monotherapy alone and without systemic antibiotics (Figure 11). This is a groundbreaking finding. Unlike commercial silver dressings or antibiotics, which often face challenges with bacterial resistance [53,54,55], the PAW–Carbopol^®^ 940 hydrogel remains highly effective and low-cost. In this regard, the system leverages ROS not only to eradicate infection but also to act as cell signalling molecules that catalyse granulation and angiogenesis. It can be manufactured using DBD technology, with only oxygen and low-power electricity. These features make the system a superior, cost-effective option for resource-limited settings.

From a physiological perspective, the controlled delivery of ROS at low concentrations mimics the natural respiratory burst of macrophages [56,57,58,59]. This microenvironment induces an “oxidative eustress” which, far from causing massive cell damage, stimulates regenerative signalling pathways that promote granulation and accelerated angiogenesis [60].

#### 2.6.3. Limitations and Future Perspectives

Despite promising results, this work raises questions for future research. Molecular biology assays are needed to quantify growth factor expression—specifically VEGF—and clarify the cell signalling pathways activated by the hydrogel [61,62]. It is also important to test the system against more complex, multispecies biofilms, including Methicillin-resistant *Staphylococcus aureus* strains [63].

This study emphasised biophysical characterisation and validation in a clinical proof-of-concept trial. The findings constitute the technical scaffolding needed for the design of large-scale, randomised, controlled clinical trials with robust statistical power.

In synthesis, this study shows the synergy between non-thermal plasma technology and Carbopol^®^ 940 matrices as an innovative biotechnological approach. The PAW–Carbopol^®^ 940 hydrogel not only stabilises ultra-short-lived ROS but modulates their delivery kinetics for therapeutic use in complex pathologies. This development stands as a robust, sustainable, and low-cost alternative to traditional antibiotics and dressings for chronic wound care.

## 3. Conclusions

This research validates the successful functionalization of a Carbopol^®^ 940 matrix with plasma-activated water (PAW), establishing an advanced reactive oxygen species (ROS) delivery system with high translational potential in biomedicine. The results demonstrate that the polymer matrix resolved the critical challenge posed by the inherent instability of plasma technology by acting as a highly efficient chemical reservoir, achieving 100% retention of O_3_ and H_2_O_2_ concentrations for 90 min. Follow-up stability assays confirmed that the matrix maintains therapeutic concentrations of H_2_O_2_ (3 ppm) for up to 45 h, ensuring prolonged activity beyond the initial application. From a structural perspective, FTIR analysis confirmed the correct ionisation of the polymer into a crosslinked polyacrylate network, while SEM characterisation revealed a highly organised nanoporous architecture (~1433 nm). This three-dimensional configuration is the determining biophysical factor that facilitates the confinement of ROS and regulates their sustained diffusion into the application bed. In terms of biological activity, the hydrogel maintained absolute microbicidal potency against *Escherichia coli*, as evidenced by a clear inhibition zone. The system operates under modulated kinetics that extended the inactivation time to 90 min, three times longer than in liquid PAW; this behaviour is fundamental to ensuring constant oxidative pressure without inducing acute cytotoxicity. Finally, the clinical validation in a patient with a diabetic foot ulcer constitutes the most significant finding of the study. Conducted under the approval of the Institutional Research Ethics Committee (UEeIM: 314/26) and registered at ClinicalTrials.gov (NCT07541196), the study demonstrates that the hydrogel, as monotherapy, achieves complete resolution of chronic lesions within 60 days by effectively eradicating infection and promoting tissue regeneration through the induction of oxidative eustress. While this preliminary case study provides compelling evidence of the hydrogel’s translational potential and biocompatibility, the ongoing controlled clinical trial will further establish the statistical significance and generalised efficacy of this platform. As a limitation of this preliminary design, it must be noted that these findings are based on a single-case observation (*n* = 1) lacking a control group; thus, comprehensive clinical trials with larger cohorts are required to establish generalised clinical efficacy and statistical significance. In conclusion, the PAW–Carbopol^®^ 940 hydrogel stands as an innovative, biocompatible, and robust therapeutic platform with disruptive potential in both regenerative medicine and the global strategy to combat antimicrobial resistance.

## 4. Materials and Methods

### 4.1. Chemical Materials and Microorganisms

Pharmaceutical-grade Carbopol^®^ 940 (Gardenia Naturals Labs, Guadalajara, Mexico) was used as the precursor agent for the polymer matrix, selected for its high-water retention capacity and biocompatibility. For plasma activation, high-purity O_2_ (99.99%, Infra, Toluca, Mexico) was used as the precursor gas to generate reactive oxygen species (ROS), specifically ozone (O_3_) and hydrogen peroxide (H_2_O_2_). Distilled and deionised water was used to produce plasma-activated water (PAW).

For the microbiological assays, the *Escherichia coli* ATCC 8739 strain was used as a model Gram-negative pathogen. Cultures were grown on Luria–Bertani (LB) medium in both solid and liquid forms. For dilutions, this were performed in sterile saline solution (0.85% *w*/*v* NaCl). The quantification of ROS was carried out using validated colourimetric and titration kits (Hanna Instruments, Woonsocket, RI, USA): HI38054 for O_3_ and HI3844-0 for H_2_O_2_.

### 4.2. Plasma-Activated Water (PAW) Production

PAW synthesis was carried out using a coaxial, continuous-flow dielectric barrier discharge (DBD) reactor (Figure 11). The system integrates an external stainless-steel electrode with a nominal diameter of 1.27 cm and 43 cm long. This electrode defines the geometry of the discharge volume. The device consists of a central tungsten electrode coated with a high-permittivity ceramic barrier. The dielectric interface is critical. It allows the formation of homogeneous microdischarges and prevents transition to an arc. This optimises energy transfer for generating reactive species in the liquid phase.

**Figure 12:**
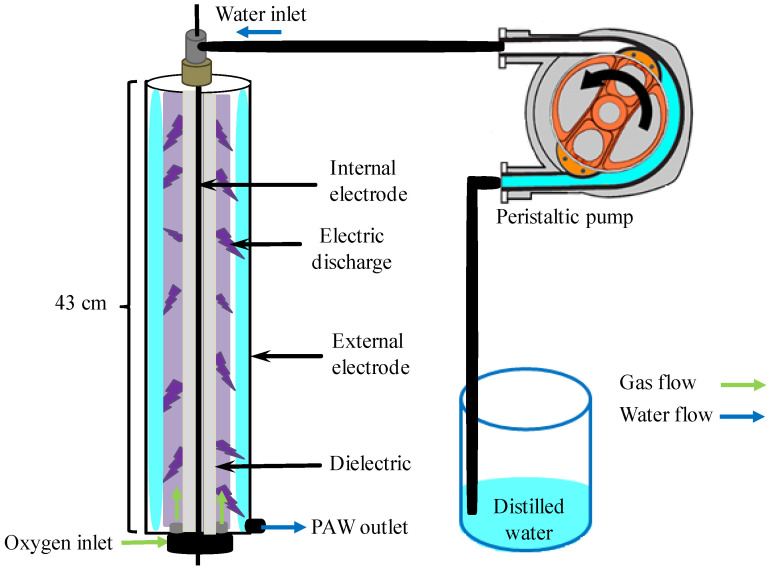
Schematic representation of the synthesis of PAW.

A total volume of 100 mL of water was processed under a constant flow regime at 100 mL/min. Plasma operating conditions included a pulsed power source with a pulse width of 20 μs, peak voltage of 15 kV, and frequency of 500 Hz. Oxygen (O_2_) was supplied at 0.5 L/min (MF5708, Siargo Ltd., Santa Clara, CA, USA). The treatment involved three complete recirculation cycles. Given the reactor geometry, the water travelled an effective activation length of 129 cm. The exposure volume per cycle was 33.77 cm^3^. This recirculation protocol ensures optimal mass transfer of chemical species between the gas and liquid phases (Figure 12).

To ensure the reproducibility of the process, all syntheses were conducted under controlled environmental conditions (22 ± 1 °C, 45 ± 5% relative humidity) with a fixed electrode gap. Inter-batch variability analysis across ten independent production cycles demonstrated high stability, with a standard deviation below 5% for both O_3_ and H_2_O_2_ concentrations. These results confirm that the standardised DBD protocol is robust and suitable for quality-controlled manufacturing of the functionalized hydrogel.

### 4.3. Hydrogel Formulation and Characterisation

#### 4.3.1. Hydrogel Synthesis and Neutralisation

**Figure 13:**
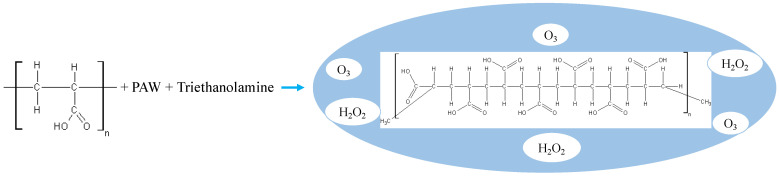
Schematic of the PAW + Carbopol^®^ 940 hydrogel formulation.

Immediately after water activation, hydrogel preparation began to preserve ROS integrity. Specifically, 1.0 g of Carbopol^®^ 940 (Figure 13) was dispersed in 100 mL of PAW with constant mechanical stirring (BT4000, Benchmark Scientific Inc., Sayreville, NJ, USA) at 100 rpm for 15 min at room temperature (25 ± 2 °C). This process initiated the hydration of the crosslinked polyacrylic acid chains, leading to the formation of an acidic colloidal dispersion.

The system’s reactivity depends on the formation of oxidising species at the interface, as described by the following reactions [64,65]:

e^−^ + H_2_O → HO^−^ + H^+^ + e^−^ (1)

H_2_O + e^−^ → H^•^ + ^•^OH + e^−^ (2)

e^−^ + O_2_ → O_2_^−^ (3)

2H^+^ + 2O_2_^−^ → O_2_ + H_2_O_2_ (4)

O_2_ + e^−^ → 2O + e^−^ (5)

O_2_ + O → O_3_ (6)

^•^OH + ^•^OH → H_2_O_2_ (7)

Prior to dispersion, the medium was observed to acidify. This occurred due to the interaction of plasma-derived acidic species. As a result, the pH (HI 9828, Hanna Instruments, RI, USA) decreased from 5.4 ± 0.2 (distilled water) to 4.2–5.1 (PAW). To induce the sol-to-gel transition, the mixture was neutralised by the dropwise addition of 10% (*v*/*v*) triethanolamine (TEA). This process promotes the ionisation of the polymer’s carboxyl (-COOH) groups to carboxylate (-COO^−^) residues. The resulting intramolecular electrostatic repulsion triggers the conformational expansion of the three-dimensional network. This structural rearrangement significantly increases the system’s viscosity and stabilises ROS within the nanoporous structure. Finally, the pH was adjusted to a physiological value (5.5 ± 0.2). This ensures biocompatibility for dermal applications.

#### 4.3.2. Quantification and Stability of Reactive Oxygen Species (ROS)

O_3_ and H_2_O_2_ were quantified in both the liquid PAW and the hydrogel matrix to evaluate the polymer’s stabilisation capacity against the reactive species of interest. The protocols are described below:

- Ozone (O_3_): It was determined by absorption spectrophotometry using a commercial kit HI38054 based on the N, N-diethyl-p-phenylenediamine (DPD) colourimetric method. The intensity of the coloured complex was correlated with ozone concentration using a visual comparison system calibrated in parts per million (ppm).
- Hydrogen peroxide (H_2_O_2_): Concentration was quantified by iodometric redox titration (HI3844-0 kit). The procedure involved stoichiometric release of iodine from potassium iodide in acid, followed by titration with sodium thiosulfate. The endpoint was determined by a colour change in a starch indicator.

Chemical stability was assessed by monitoring ROS levels over 14 days. Samples were stored at 4 ± 1 °C (05LCEETSA, Thermo Scientific, Waltham, MA, USA), simulating standard clinical storage conditions.

#### 4.3.3. Morphological Characterisation by Scanning Electron Microscopy (SEM)

The surface morphology and nanoarchitecture of the hydrogel were examined using a scanning electron microscope (SEM) model JSM-5900 (JEOL, Tokyo, Japan). To preserve the integrity of the three-dimensional network and minimise polymer shrinkage or structural collapse induced by conventional drying, the samples were previously lyophilised in a Heto Drywinner (Heto-Holten, Allerød, Denmark), thus ensuring the observation of the morphology in its quasi-native state.

The analysis was performed at an accelerating voltage of 20 kV, a working distance of 10 mm, and under high-vacuum conditions. To mitigate the effects of electrostatic charge and optimise image resolution, the lyophilised samples were coated with an ultrathin gold layer using a sputtering system (Desk II, Denton Vacuum, Moorestown, NJ, USA) for 40 s.

This characterisation enabled evaluation of the polymer matrix homogeneity, network interconnectivity, and pore size distribution, as well as the identification of aggregates. These morphological properties are critical parameters for the hydrogel’s functionality, as they directly govern the diffusion mechanisms and sustained release kinetics of the encapsulated ROS.

### 4.4. In Vitro Evaluation of Antibacterial Activity

#### 4.4.1. Preparation of the Bacterial Inoculum

The *Escherichia coli* (*E. coli*) strain was cultured in Luria–Bertani (LB) broth at 37 °C for 24 h with shaking until reaching the stationary phase. Cells were collected by centrifugation at 5000 rpm for 10 min, the supernatant discarded, and the cell pellet resuspended in sterile distilled water. This washing step was repeated twice to remove residual culture medium that could interfere with bacterial inactivation.

Cell concentration was standardised to 10^7^ CFU/mL by volumetric dilution, adjusting 200 µL of inoculum with 1800 µL sterile distilled water. Optical density at 600 nm was measured by spectrophotometry to confirm an initial concentration of 1 × 10^7^ CFU/mL, ensuring reproducibility in subsequent assays.

#### 4.4.2. Contact Protocol and Determination of Antimicrobial Efficacy

To evaluate the bacterial inactivation, 1800 µL of the hydrogel was mixed with 200 µL of the standardised inoculum (10^7^ CFU/mL), achieving a final effective concentration of 1 × 10^6^ CFU/mL in the test system. To isolate the effect of ROS and validate their mechanism of action, an experimental control was established consisting of Carbopol^®^ 940 hydrogel prepared with distilled water (without plasma treatment) and neutralised with TEA to a pH of 5.5 ± 0.2. This control is critical to rule out any intrinsic antimicrobial activity of the polymer or variations due to the medium’s acidity.

During the test period, the mixtures were continuously mechanically agitated to ensure homogeneous interaction between the bacterial cells and the ROS gradually released from the polymer matrix. To monitor cell death kinetics, samples were collected at 10, 30, 60, and 90 min.

At the end of each interval, aliquots were extracted and subjected to vigorous vortexing to ensure the mechanical dissociation of bacterial cells from the Carbopol^®^ 940 micro-network. This step was followed by serial decimal dilutions performed in sterile saline solution (0.85% *w*/*v* NaCl). Next, 100 µL of each dilution was plated onto LB agar plates using the surface spreading technique with a sterile Drigalsky spatula. The plates were incubated at 37 °C for 24 h, after which viable cell colonies were counted. The combination of continuous agitation during treatment and vigorous vortexing before plating ensures that the CFU counts accurately reflect the total viable population, preventing underestimation due to bacteria being trapped within the hydrogel matrix.

Finally, the hydrogel efficacy was evaluated by counting CFU/mL and determining the percentage of bacterial inactivation:

$$Percentage\;of\;inactivation=\left(1-\left(\frac{CFU\;treated}{CFU\;control}\right)\right)100\%$$

where CFU treated are the CFU counted after applying the treatment, and CFU control are the CFU without applying the treatment. To ensure the statistical robustness and reproducibility of the findings, ten independent experiments were performed for each trial and each one was realised by triplicate.

### 4.5. Ethical Considerations and Consent

The clinical case included in this research was conducted in strict compliance with the ethical principles established in the Declaration of Helsinki of the World Medical Association. The intervention protocol, entitled “Healing of Chronic Wounds through the Application of Non-Thermal Plasma,” was evaluated and approved by the Health Research and Research Ethics Committee of the ISSEMyM Medical Centre, Lic. Arturo Montiel Rojas, as recorded in document number 203F 39101/1000/CMI/620/2017, derived from session number 175.

Prior to the start of treatment, written informed consent was obtained from the patient after a detailed explanation of the study’s nature, potential benefits, and minimal associated risks. This document included express authorisation for the photographic recording and dissemination of images for strictly scientific and academic purposes in specialised publications. In compliance with current personal data protection regulations, anonymity and confidentiality were guaranteed at all times, eliminating any identifying features from the submitted graphic material.

### 4.6. Statistical Analysis

All chemical quantification experiments (ROS stability) and microbiological assays were performed in triplicate (*n* = 3). Results are expressed as mean ± standard deviation. Statistical significance was assessed using a one-way ANOVA, with *p*-values < 0.05 considered significant. Data processing was carried out using OriginPro software ver. 8.5.0 SR1 (OriginLab Corporation, Northampton, MA, USA).

## Data Availability

All data generated or analysed during this study are included in this published article. However, in compliance with institutional regulations on patient confidentiality and data management policies, the complete raw, anonymised individual participant data (IPD) underlying the results of this article are available upon reasonable request addressed to the corresponding author. Access will be granted upon a formal data-sharing agreement, approval by the Institutional Ethics Committee, and confirmation of the request’s justification. Data access will be subject to a strict protocol of data anonymisation and will not be granted for commercial purposes.

## Abbreviations

The following abbreviations are used in this manuscript:

PAW	Plasma-Activated Water
NTP	Non-Thermal Plasma
ROS	Reactive Oxygen Species
DBD	Dielectric Barrier Discharge
DFU	Diabetic Foot Ulcer
AMR	Antimicrobial Resistance
RNS	Reactive Nitrogen Species
FTIR	Fourier Transform Infrared Spectroscopy
SEM	Scanning Electron Microscopy
TEA	Triethanolamine
